# Medical and Philosophical News

**Published:** 1781-01

**Authors:** 


A paper has been lately communicated to the Royal Society by Richard Kirwan, Esq. F.R.S. describing the means of ascertaining the specific quantity of pure acid contained in any acid salt. In this paper the ingenious author has particularly determined the exact quantity of fixed air contained in different substances. By combining algebraical calculations with chemistry (a method that seems to be equally new and ingenious) he finds himself able to ascertain the 2500th part of a grain. The same philosopher is at present prosecuting experiments with a view to determine the specific proportion of phlogiston contained in the different metals.

M. Achard has lately communicated to the Royal Academy of Sciences at Berlin, a new method of exciting a degree of heat much superior to any hitherto produced, and this by means of a ſmall quantity of charcoal, or any other combuſtible ſubſtance. At the ſame time he read the deſcription of a machine for dephlogiſticating the air of apartments, and of courſe rendering them fitter for reſpiration.

